# Early Dynamic ^18^F-FDG PET/CT SUVmax and Its Association with Pathological Stage and Tumor Grade in Bladder Cancer: A Prospective Exploratory Study

**DOI:** 10.3390/medicina62102021

**Published:** 2026-10-19

**Authors:** Barış Saylam, Berfin Temelli, Ali Nebioğlu, Ozan Efesoy, Murat Bozlu

**Affiliations:** 1 Department of Urology, Mersin City Training and Research Hospital, 33240 Mersin, Turkey; alinebioglu90@gmail.com (A.N.); oefesoy@yahoo.com (O.E.); 2 Department of Nuclear Medicine, Mersin City Training and Research Hospital, 33240 Mersin, Turkey; berfintemel@hotmail.com; 3 Department of Urology, Mersin University Faculty of Medicine, 33343 Mersin, Turkey; muratbozlu@yahoo.com

**Keywords:** bladder cancer, ^18^F-FDG PET/CT, early dynamic imaging, standardized uptake value, non-invasive staging

## Abstract

*Background and Objectives:* Accurate preoperative staging and grade assessment are crucial for optimal treatment selection in bladder cancer. This prospective study evaluated multi-timepoint Fluorine-18 fluorodeoxyglucose (^18^F-FDG) positron emission tomography/computed tomography (PET/CT) imaging for predicting pathological stage and tumor grade. *Materials and Methods:* Seventy-two patients with bladder carcinoma underwent ^18^F-FDG PET/CT including early dynamic (0–2, 2–4, 8–10 min), standard (60 min), and late (~120 min) imaging following a standardized furosemide hydration protocol. Maximum standardized uptake values (SUVmax) were correlated with histopathological findings. Diagnostic performance was evaluated using receiver operating characteristic (ROC) curve analysis with Youden index for optimal cut-offs. *Results:* The cohort comprised 72 patients (84.7% male, mean age 64.8 ± 10.7 years) with pathological stages Ta (36.1%), CIS (15.3%), T1 (31.9%), and T2 (16.7%). High-grade tumors demonstrated significantly higher SUVmax at early dynamic imaging (0–2 min: 1.50 vs. 1.00, *p* = 0.028) and late imaging (11.40 vs. 9.50, *p* = 0.045) compared to low-grade tumors. Early dynamic imaging at 0–2 min achieved the highest, although modest, diagnostic performance for high-grade prediction (area under the curve (AUC) = 0.652) and muscle-invasive disease prediction (AUC = 0.669), with optimal cut-offs of SUVmax = 1.40 and 1.80, respectively. Early dynamic SUVmax correlated significantly with tumor grade (ρ = 0.261, *p* = 0.027) and tumor size (ρ = 0.424, *p* < 0.001). The negative predictive value for muscle-invasive disease was 90.4%, but sensitivity was only 58.3%. *Conclusions:* Early dynamic ^18^F-FDG PET/CT imaging at 0–2 min showed the highest, although modest, diagnostic performance among the evaluated time points for predicting tumor grade and muscle invasion. The identified SUVmax cut-offs are exploratory and require external validation in larger multicenter studies before clinical use. *Trial registration:* Not registered.

## 1. Introduction

Fluorine-18 fluorodeoxyglucose (^18^F-FDG) positron emission tomography/computed tomography (PET/CT) is a non-invasive imaging modality that provides whole-body imaging in many types of cancer [1]. It has gained increasing popularity in cancer assessment based on the hypermetabolism observed in malignant cells [2]. It provides comprehensive information about metabolic and structural changes in the body by combining the functional information provided by ^18^F-FDG PET/CT with the anatomical details of computed tomography (CT). In particular, ^18^F-FDG PET/CT provides information based on glucose uptake and detects cells with high uptake (e.g., neoplastic cells that utilize glucose more) [3]. It is well known that the ability of ^18^F-FDG PET/CT to detect tumoral tissue is based on increased expression of cell membrane glucose transporter 1 (GLUT1) and increased hexokinase II enzyme activity in tumor cells [4].

^18^F-FDG PET/CT uptake was determined as the maximum standardized uptake value (SUVmax) in the lesion localized by cystoscopy or the most active lesion. In other malignancies, such as ovarian carcinoma, SUVmax values have been shown to correlate with the grade and histology of the tumor [5]. Similar findings have been reported in many other types of cancer. For example, high-grade salivary malignancies have been found to have higher ^18^F-FDG uptake than low- or intermediate-grade salivary tumors [6]. Similarly, there are studies showing that increased glucose metabolism determined by SUV is associated with tumor grade and survival in gliomas [7]. A similar relationship has been demonstrated for other cancers, such as lung and esophageal cancers, in which FDG uptake has been associated with prognosis and treatment response [8,9]. High ^18^F-FDG uptake is associated with aggressiveness and decreased survival. Tumor histological grade may be estimated from ^18^F-FDG uptake, which may help in treatment planning.

The standard method for diagnosing bladder cancer (BC) is based on direct visualization of the bladder during cystoscopy followed by histopathological examination of the removed specimen. Both treatment and prognosis of BC are based on the tumor–node–metastasis (TNM) stage. At diagnosis, 75–85% of patients have non-muscle-invasive bladder cancer (NMIBC), and 15–25% have muscle-invasive bladder cancer (MIBC) [10]. EAU guidelines still recommend CT and magnetic resonance imaging (MRI) for MIBC staging, but the EAU guidelines also note that ^18^F-FDG PET/CT is increasingly used in clinical practice for LN staging [11].

The role of ^18^F-FDG in detecting localized BC is limited due to the difficulty in distinguishing radiotracer activity excreted in the urine from tumor uptake in the bladder. Protocols such as post-voiding images, furosemide injection before image acquisition, retrograde bladder irrigation with a catheter, and delayed image acquisition after diuretics and oral hydration have been implemented to increase the sensitivity of PET [12]. The relationship between SUVmax value during ^18^F-FDG PET/CT of BC and the aggressiveness of the disease has been investigated in a previous study, but studies with a larger number of patients are needed in the literature [13].

The aim of this prospective study is to contribute to the literature by evaluating the relationship between SUVmax values and the grade and pathological T stage of bladder tumors using early dynamic pelvic imaging and standard ^18^F-FDG PET/CT.

## 2. Materials and Methods

**Figure 1:**
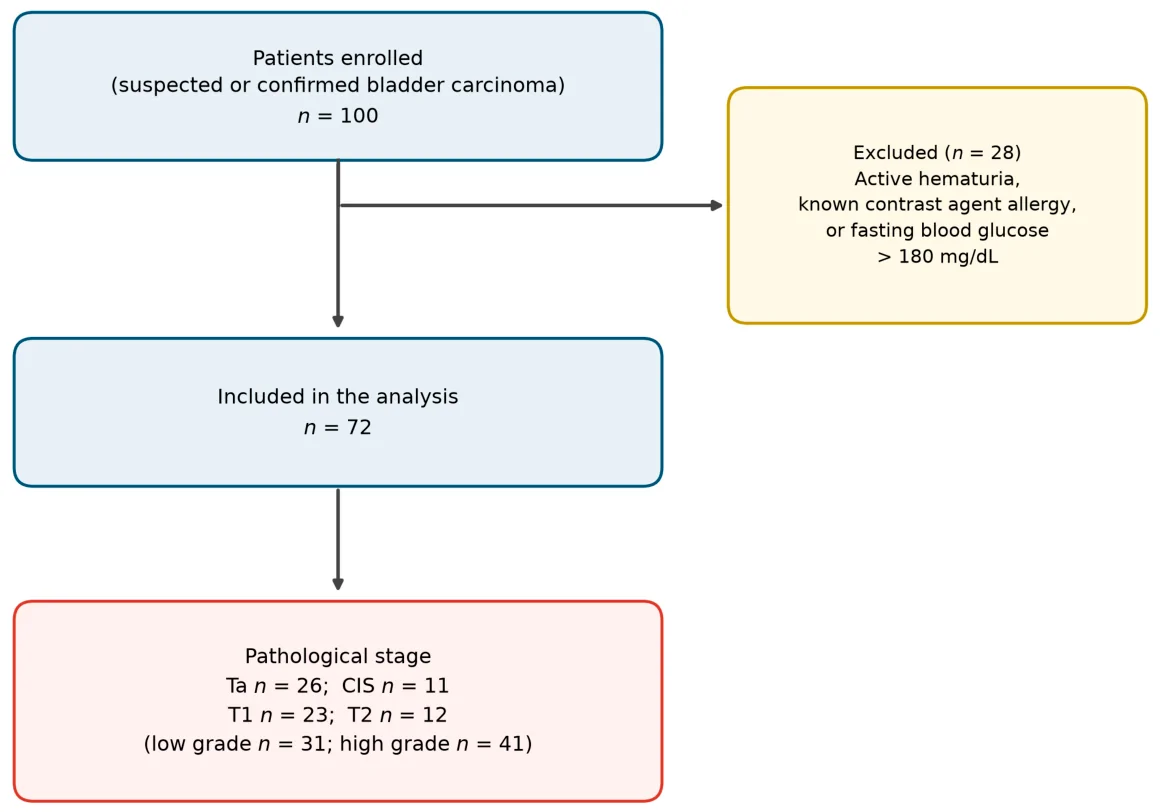
Flow Diagram of Patient Enrollment and Inclusion.

This prospective study was performed between May 2024 and June 2025 at Mersin City Training and Research Hospital. Approval was obtained from the Mersin University Ethics Committee for the study (2024/421). All patients who participated in the study were informed about the study and gave written consent. Following the initial enrollment of 100 patients, the application of the exclusion criteria (active hematuria, known contrast agent allergy, or fasting blood glucose >180 mg/dL; Figure 1) resulted in the inclusion of 72 patients with suspected or newly diagnosed primary bladder carcinoma scheduled for TURBT (none had received prior intravesical bacillus Calmette–Guérin (BCG) or systemic chemotherapy) in the study. All patients were required to fast for at least 6 h prior to the PET/CT scan. Blood sugar levels were measured in all patients before PET/CT scanning. After collecting the patients’ clinical histories and demographic information, each patient was placed in a single-person lead-shielded room. Patients were instructed not to empty their bladders upon arrival at the nuclear medicine department. Dynamic scans were performed using a PET/CT scanner (GE Discovery IQ Gen; GE Healthcare, Milwaukee, WI, USA). A low-dose CT scan was performed first, followed by the administration of ^18^F-FDG. The FDG dose was administered intravenously at a rate of approximately 5.3 MBq per kilogram, not exceeding a maximum of 518 MBq. Approximately 30 min after the FDG injection, patients were administered 10 mg of furosemide intravenously in 250 mL of physiological saline. This hydration protocol was chosen, as it had shown the best results in a previous study [14]. Subsequently, patients were asked to empty their bladders frequently and to refrain from voiding for 10–15 min before the scan. Standard whole-body PET/CT imaging was completed in 8 bed positions, with each position taking 2 min, from the vertex to the thigh. Patients were instructed to use shallow breathing between the acquisition of PET and CT images, to minimize misregistration. Additionally, delayed scans were performed approximately 120 min after the FDG injection, during which the pelvic region was reimaged with the bladder full.

PET/CT images were analyzed on a GE AW4.7 workstation (GE Healthcare, Milwaukee, WI, USA) by a single nuclear medicine specialist with more than 10 years of experience in oncologic PET/CT, who was aware of the clinical indication but blinded to the histopathological findings, which were obtained only after image interpretation. After CT attenuation correction, transverse, coronal, and sagittal plane images, as well as maximum intensity projection (MIP) images, were evaluated. The early dynamic pelvic acquisition started at the time of ^18^F-FDG injection and consisted of six consecutive 2-min frames (12 min in total); images were reconstructed using the vendor’s iterative ordered-subset expectation maximization algorithm (VUE Point HD) with resolution recovery and CT-based attenuation, scatter, and randoms corrections (4 iterations, 12 subsets, 4-mm Gaussian post-filter), and a volume of interest (VOI) was delineated on the fused PET/CT images around the bladder lesion while avoiding the urinary activity in the bladder lumen. The VOI was drawn on the first (0–2 min), second (2–4 min), and fifth (8–10 min) frames and re-adjusted on each analyzed frame, and weight-corrected SUVmax values were measured. The same analyses were performed for lesions within the bladder during standard whole-body PET/CT scans and late pelvic imaging. The dimensions of the lesion within the bladder were measured in the standard whole-body PET/CT scan. In addition, standard PET/CT scans were evaluated for the presence of lymph nodes and metastases. The retention index was calculated as [(SUVmax at ~120 min − SUVmax at 60 min)/SUVmax at 60 min] × 100. SUVmax was not corrected for partial-volume effects, and SUVmean, SUVpeak, and tumor-to-background ratios were not analyzed.

The ^18^F-FDG PET/CT uptake was determined as the SUVmax of the index lesion. In patients with multiple tumors, the lesion to be measured was identified from the cystoscopic findings (tumor location and size recorded on a bladder diagram by the urologist) and matched to the PET/CT lesion by its position in the bladder wall; when correspondence could not be established, the most FDG-avid lesion was analyzed (*n* = 6 of the 35 patients with multiple tumors). For carcinoma in situ without a discrete mass, the VOI was placed over the most focally avid bladder-wall region corresponding to the cystoscopically suspicious or mapping-biopsy site. The reader had access to the cystoscopic localization but not to any histopathological result. The grade and invasion of the tumor were ascertained from the histopathological examination of tissues using the World Health Organization/International Society of Urological Pathology (WHO/ISUP) revised consensus classification of 2004, following transurethral resection of bladder tumor (TURBT). PET/CT was performed before TURBT in all patients, and TURBT specimens were evaluated by the institutional pathology department; repeat TURBT or radical cystectomy specimens were not systematically available.

### Statistical Analysis

All statistical analyses were performed using Python 3.12 (Python Software Foundation, Wilmington, DE, USA) with pandas (v2.3.3), numpy (v2.3.3), scipy (stats module), scikit-learn (metrics module), and scikit-posthocs for post hoc tests. Continuous variables were expressed as mean ± standard deviation (SD) for normally distributed data and as median (interquartile range, IQR) for non-normally distributed data. The Shapiro–Wilk test was used to assess normality. Categorical variables were presented as numbers and percentages. The Kruskal–Wallis H test was used to compare SUV values across pathological stages, with Dunn’s post hoc test and Bonferroni correction for pairwise comparisons. The Mann–Whitney U test was used for two-group comparisons. Spearman’s rank correlation coefficient was calculated to assess relationships between SUV parameters and tumor grade (low = 0, high = 1) and tumor size. Pathological stage was not entered into rank correlation analysis as a numerically coded variable, because CIS does not occupy an ordered position between Ta and T1; stage groups (Ta, CIS, T1, T2) were compared with the Kruskal–Wallis test, and muscle-invasive disease (T2 vs. Ta/CIS/T1) was analyzed as a binary outcome. Receiver operating characteristic (ROC) curve analysis was performed to evaluate diagnostic performance, with optimal cut-off values determined by the Youden index. The 95% confidence intervals of the AUCs were estimated with the DeLong method, those of sensitivity, specificity, PPV, and NPV with Wilson score intervals, and those of likelihood ratios with the log method. The effect size of the Mann–Whitney U test was expressed as the rank-biserial correlation. In a sensitivity analysis, the grade comparison (Mann–Whitney U test), the Spearman correlations, and the ROC analyses at 0–2 min (with cut-offs re-derived) were repeated after excluding patients in whom the PET/CT lesion could not be matched to the cystoscopic lesion. Multiple time points and outcomes were analyzed without correction for multiplicity, and all analyses are therefore exploratory. Logistic regression and multivariable adjustment (e.g., for tumor size) were not performed because of the small number of T2 events. *p* < 0.05 was considered statistically significant.

## 3. Results

A total of 72 patients with bladder carcinoma were included in this prospective study (Figure 1). The cohort comprised predominantly male patients (*n* = 61, 84.7%) with a mean age of 64.8 ± 10.7 years (range: 31–84 years). The baseline demographic, clinical, and tumor characteristics stratified by pathological stage are presented in Table 1. No significant differences were observed among pathological stage groups regarding age (*p* = 0.590), gender distribution (*p* = 0.956), tumor size (*p* = 0.577), or tumor number (*p* = 0.734).

Regarding pathological stage distribution, 26 patients (36.1%) had Ta disease, 11 (15.3%) had carcinoma in situ (CIS), 23 (31.9%) had T1 tumors, and 12 (16.7%) had muscle-invasive T2 disease. The mean tumor size was 2.84 ± 1.32 cm, with approximately half of the patients presenting with solitary tumors (*n* = 37, 51.4%) and the remainder with multiple lesions (*n* = 35, 48.6%). Histopathological grading revealed that high-grade tumors were more prevalent than low-grade tumors (*n* = 41, 56.9% vs. *n* = 31, 43.1%, respectively). A highly significant association was observed between pathological stage and tumor grade (*p* < 0.001), with all Ta lesions classified as low-grade, while all CIS and T2 tumors were high-grade. Among T1 tumors, 78.3% were high-grade and 21.7% were low-grade. Stage and grade were therefore closely related in this cohort, so their associations with SUVmax cannot be considered independent of each other.

**Figure 2:**
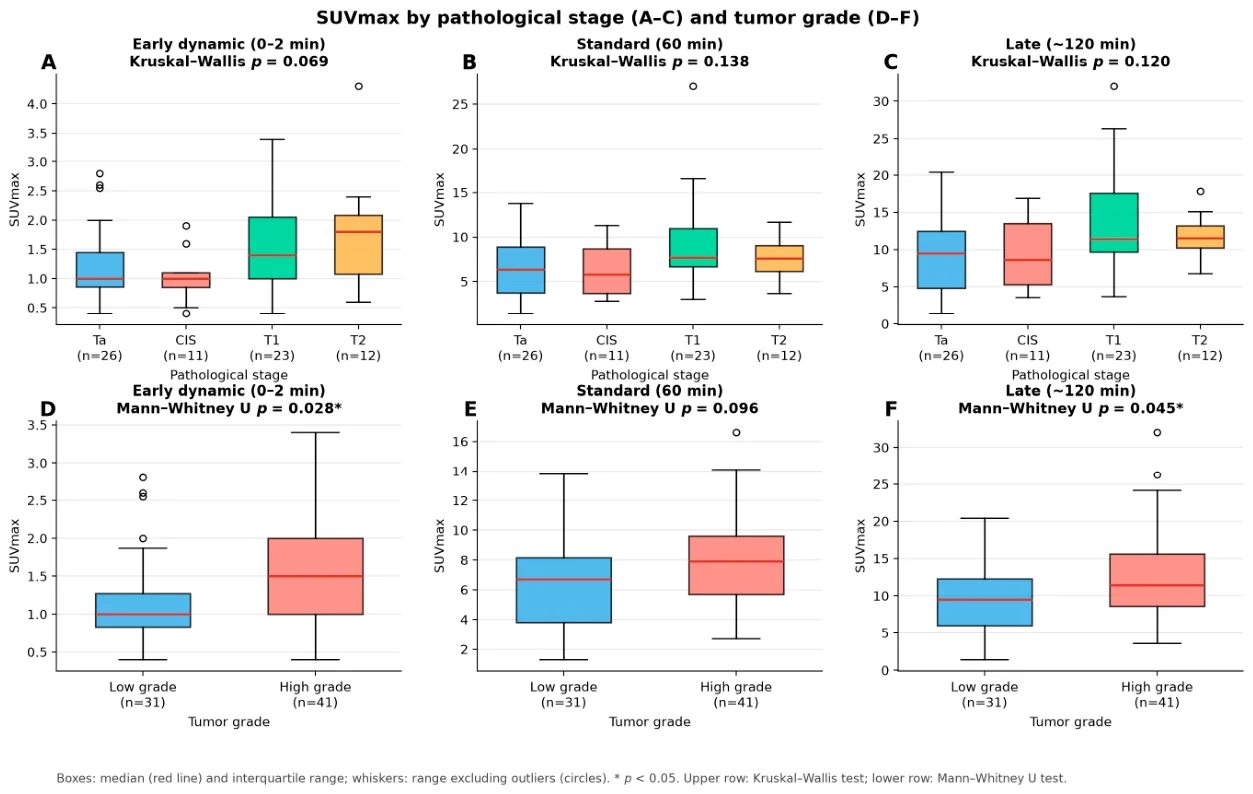
^18^F-FDG PET SUVmax Values by Pathological Stage and Tumor Grade. (**A**–**C**) SUVmax by pathological stage (Ta, CIS, T1, T2) at early dynamic (0–2 min), standard (60 min), and late (~120 min) imaging, respectively; (**D**–**F**) SUVmax by tumor grade (low vs. high) at the same time points.

SUVmax measurements at multiple time points (0–2 min, 2–4 min, 8–10 min, 60 min, and ~120 min) were compared across pathological stages (Table 2A, Figure 2A–C). All SUV parameters showed a non-normal distribution by Shapiro–Wilk testing (all *p* < 0.05), warranting non-parametric statistical analyses. SUVmax values tended to be higher in T1 and T2 tumors than in Ta tumors, but these differences were not statistically significant in the Kruskal–Wallis test.

Figure 2 shows SUVmax by pathological stage (A–C) and tumor grade (D–F) at 0–2, 60, and ~120 min. SUVmax tended to be higher in T1 and T2 than in Ta and CIS tumors, but stage-wise differences were not significant at any time point. High-grade tumors had significantly higher SUVmax than low-grade tumors at 0–2 min and ~120 min, whereas the difference at 60 min was not significant.

At 0–2 min, stage-wise differences in SUVmax were closest to statistical significance (*p* = 0.069; Figure 2A), with higher medians in T1 (1.40) and T2 (1.80) than in Ta (1.00) and CIS (1.00). No significant stage-wise differences were found at 2–4 min (*p* = 0.302) or 8–10 min (*p* = 0.697).

Stage-wise differences were also not significant at 60 min (*p* = 0.138; Figure 2B) or ~120 min (*p* = 0.120; Figure 2C), and the retention index did not vary significantly across stages (*p* = 0.540; Table 2A).

When stratified by tumor grade, high-grade tumors had higher SUVmax values than low-grade tumors at several time points (Table 2B, Figure 2D–F). The difference was statistically significant at 0–2 min (median 1.50 vs. 1.00, *p* = 0.028; rank-biserial correlation = 0.304), corresponding to a small-to-moderate effect size (Figure 2D).

At 2–4 min, the difference was not significant (*p* = 0.060; rank-biserial correlation = 0.260), and at 8–10 min there was no difference between grades (*p* = 0.763).

At 60 min, high-grade tumors showed a non-significant trend toward higher values (median 7.90 vs. 6.70, *p* = 0.096; Figure 2E). At ~120 min, the difference was statistically significant (median 11.40 vs. 9.50, *p* = 0.045; rank-biserial correlation = 0.279; Figure 2F). The retention index did not differ between grades (*p* = 0.484).

**Figure 3:**
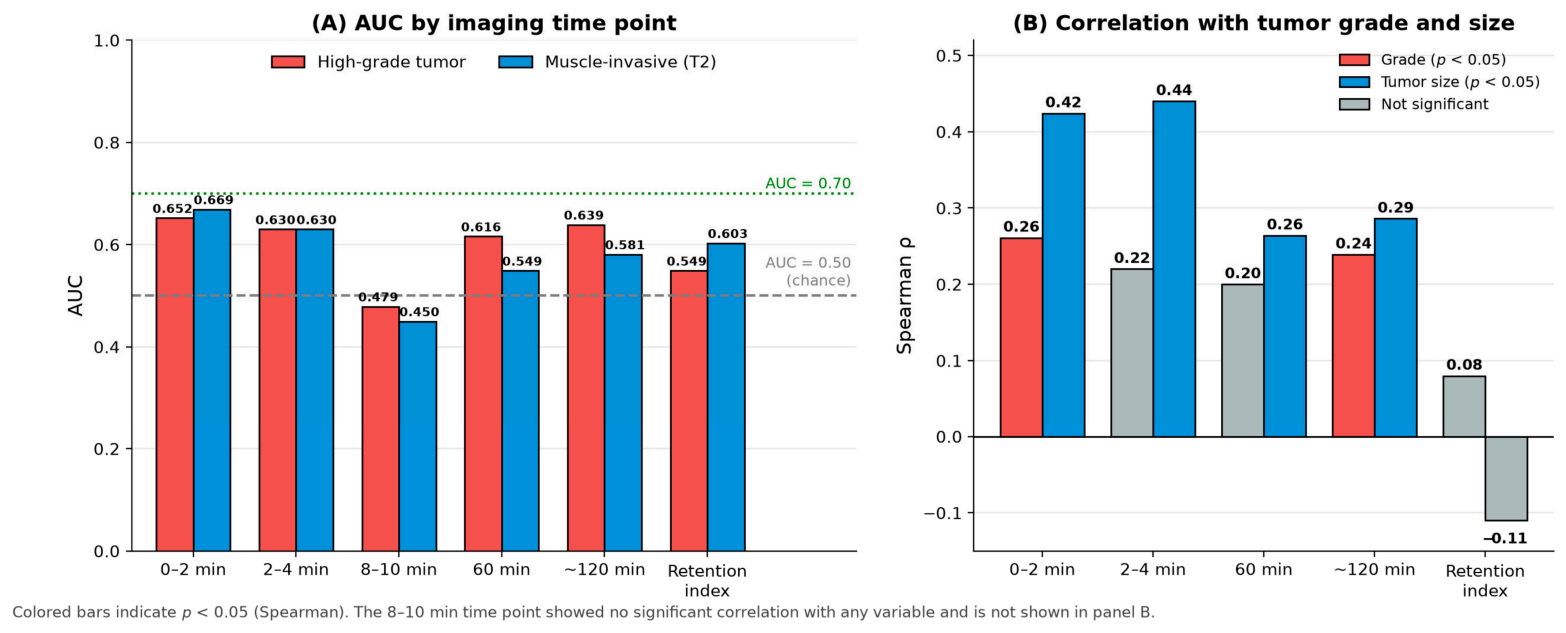
Diagnostic Performance and Correlation Analysis of ^18^F-FDG SUV Parameters.

Spearman’s rank correlation analysis revealed significant associations between SUV parameters and tumor grade and tumor size (Figure 3B). Early dynamic SUVmax at 0–2 min showed a significant positive correlation with tumor grade (ρ = 0.261, *p* = 0.027), and late imaging SUVmax showed a similar correlation (ρ = 0.239, *p* = 0.043). Stage was not analyzed by rank correlation because CIS cannot be placed on an ordinal scale between Ta and T1; stage-wise comparisons are reported in Table 2A.

Figure 3 summarizes the area under the curve (AUC) values (Figure 3A) and the Spearman correlations of SUVmax with tumor grade and size (Figure 3B). The 0–2 min time point had the highest AUCs (0.652 for high-grade tumors and 0.669 for muscle-invasive disease), whereas the 8–10 min time point performed worst (AUC = 0.479 and 0.450, respectively). Correlations with grade were weak (ρ = 0.24–0.26 for the significant ones), and correlations with tumor size were the strongest (ρ = 0.42–0.44 at 0–2 and 2–4 min).

Notably, early dynamic SUV measurements at 0–2 and 2–4 min demonstrated the strongest correlations with tumor size (Figure 3B), with correlation coefficients of ρ = 0.424 (*p* < 0.001) and ρ = 0.440 (*p* < 0.001), respectively. Standard imaging at 60 min (ρ = 0.264, *p* = 0.025) and late imaging (ρ = 0.286, *p* = 0.015) also showed significant but weaker correlations with tumor size. In contrast, SUV measurements at 8–10 min showed no significant correlation with any clinical variable, and the retention index demonstrated weak correlations without statistical significance.

**Figure 4:**
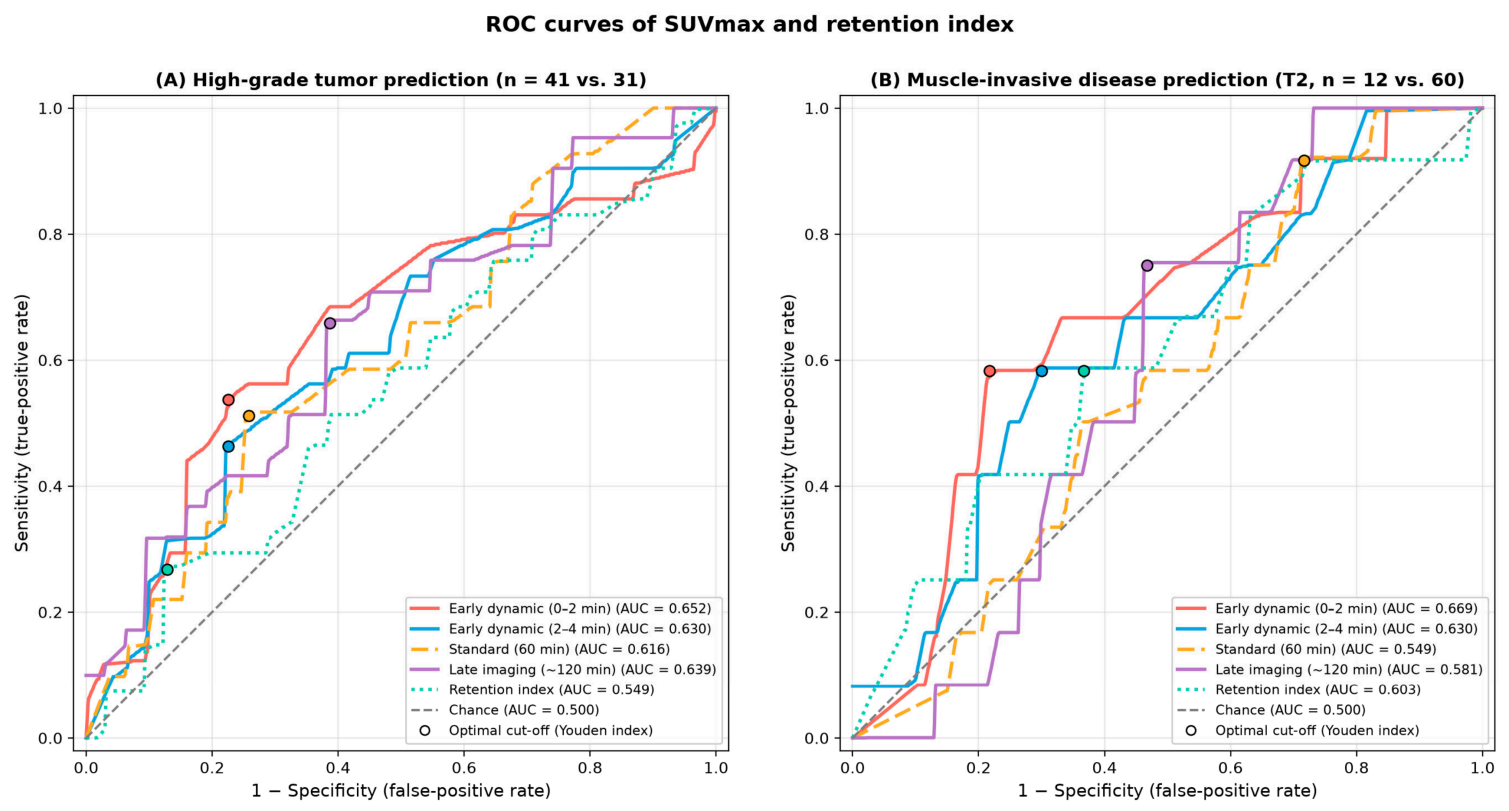
ROC Curves for High-Grade and Muscle-Invasive Disease Prediction.

ROC curve analysis was performed to evaluate the diagnostic accuracy of various SUV parameters in distinguishing high-grade from low-grade bladder tumors (Table 3A, Figure 4A). Early dynamic imaging at 0–2 min yielded the highest area under the curve (AUC = 0.652, 95% CI: 0.526–0.778), indicating modest diagnostic performance. The optimal cut-off value determined by the Youden index was 1.40, providing a sensitivity of 53.7% and specificity of 77.4%, with positive and negative predictive values of 75.9% and 55.8%, respectively.

Figure 4 shows the ROC curves for the prediction of high-grade tumors (Figure 4A) and of muscle-invasive disease (T2 vs. Ta/CIS/T1; Figure 4B). Filled circles indicate the optimal cut-off points determined by the Youden index.

Early dynamic imaging at 2–4 min showed slightly lower performance (AUC = 0.630, 95% CI: 0.502–0.758), with an optimal cut-off of 2.10 yielding sensitivity of 46.3% and specificity of 77.4%. Late imaging at approximately 120 min demonstrated an AUC of 0.639 (95% CI: 0.512–0.766), with an optimal cut-off of 10.20 providing sensitivity of 65.9% and specificity of 61.3%. Standard imaging at 60 min showed an AUC of 0.616 (95% CI: 0.486–0.746), while the retention index demonstrated poor discriminative ability (AUC = 0.549, 95% CI: 0.415–0.683).

For the prediction of muscle-invasive disease (T2 vs. Ta/CIS/T1), early dynamic imaging at 0–2 min again showed the highest AUC (Table 3B, Figure 4B). This parameter achieved an AUC of 0.669 (95% CI: 0.489–0.849), representing modest diagnostic accuracy; the lower confidence limit was below 0.5, so discrimination better than chance cannot be established in this small T2 subgroup. The optimal cut-off value of 1.80 provided a sensitivity of 58.3% and specificity of 78.3%, with a negative predictive value of 90.4%. This value should be interpreted with caution because of the low prevalence of T2 disease in this cohort (12/72) and the low sensitivity (58.3%); five of the 12 T2 tumors would have been missed. The 95% confidence intervals and likelihood ratios for the 0–2 min cut-offs are given in Table 4.

Early dynamic imaging at 2–4 min yielded an AUC of 0.630 (95% CI: 0.448–0.812), with an optimal cut-off of 2.20 providing sensitivity of 58.3% and specificity of 70.0%. Late imaging showed only modest performance (AUC = 0.581, 95% CI: 0.397–0.765), while standard imaging at 60 min demonstrated poor discriminative ability (AUC = 0.549, 95% CI: 0.366–0.732). The retention index showed slightly better performance for muscle-invasive prediction (AUC = 0.603, 95% CI: 0.420–0.786) compared to its performance in grade prediction.

Across all time points, early dynamic imaging at 0–2 min had the highest AUC for both muscle-invasive disease (0.669) and high-grade tumors (0.652), although the 95% CIs overlapped widely between time points. Early dynamic imaging (0–2 and 2–4 min) also showed the strongest correlations with tumor size. For muscle-invasive disease, the 2–4 min time point ranked second, and the 8–10 min time point performed worst for both outcomes. Standard and late imaging showed intermediate performance, with late imaging performing better for grade than for muscle-invasive disease.

In a sensitivity analysis excluding the 6 patients with ambiguous lesion correspondence (*n* = 66 analyzed), SUVmax at 0–2 min remained higher in high-grade than in low-grade tumors (median 1.50 vs. 1.00, *p* = 0.035), the Spearman correlations with grade and tumor size were ρ = 0.25 (*p* = 0.045) and ρ = 0.41 (*p* < 0.001), and the AUCs at 0–2 min were 0.648 for high-grade tumors and 0.661 for muscle-invasive disease (re-derived cut-offs 1.40 and 1.80, respectively). The findings were therefore essentially unchanged after excluding these patients.

**Figure 5:**
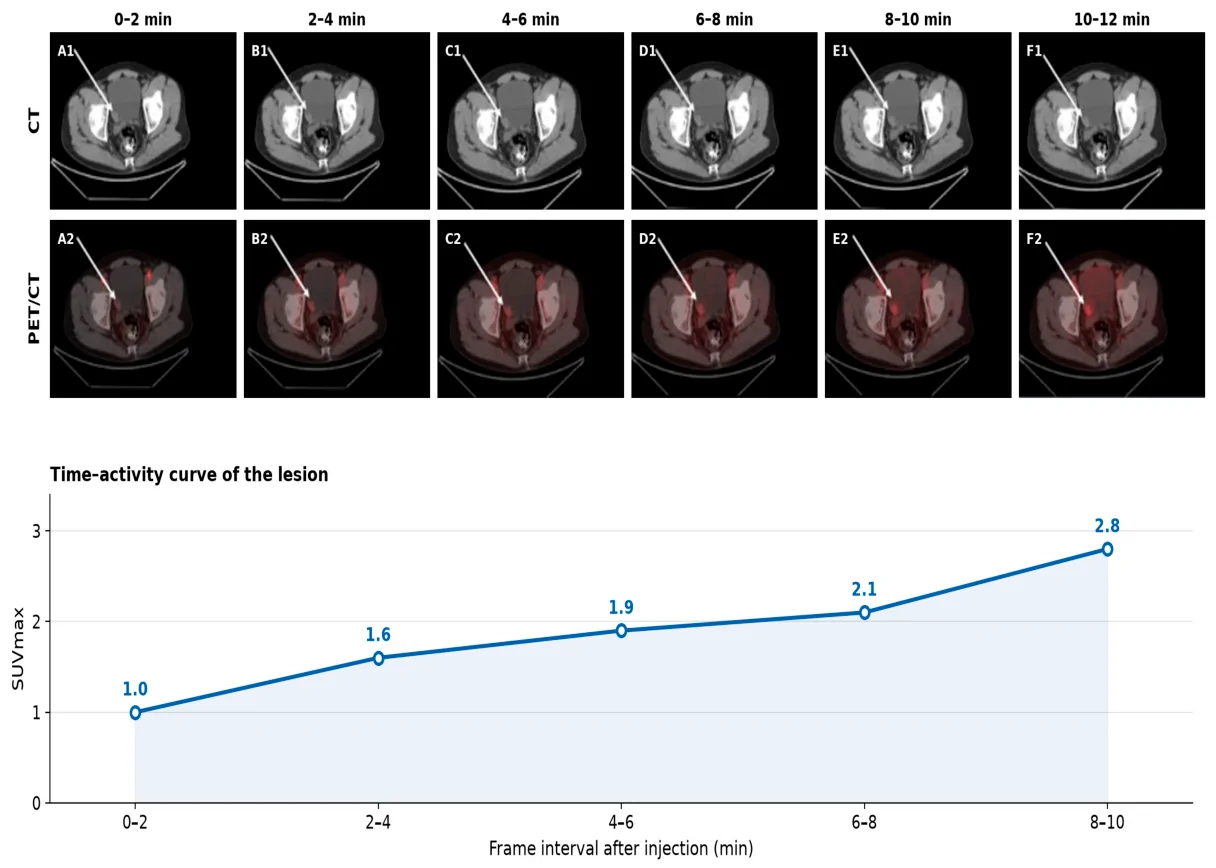
Representative Case—Low-Grade Non-Muscle-Invasive Bladder Cancer.

**Figure 6:**
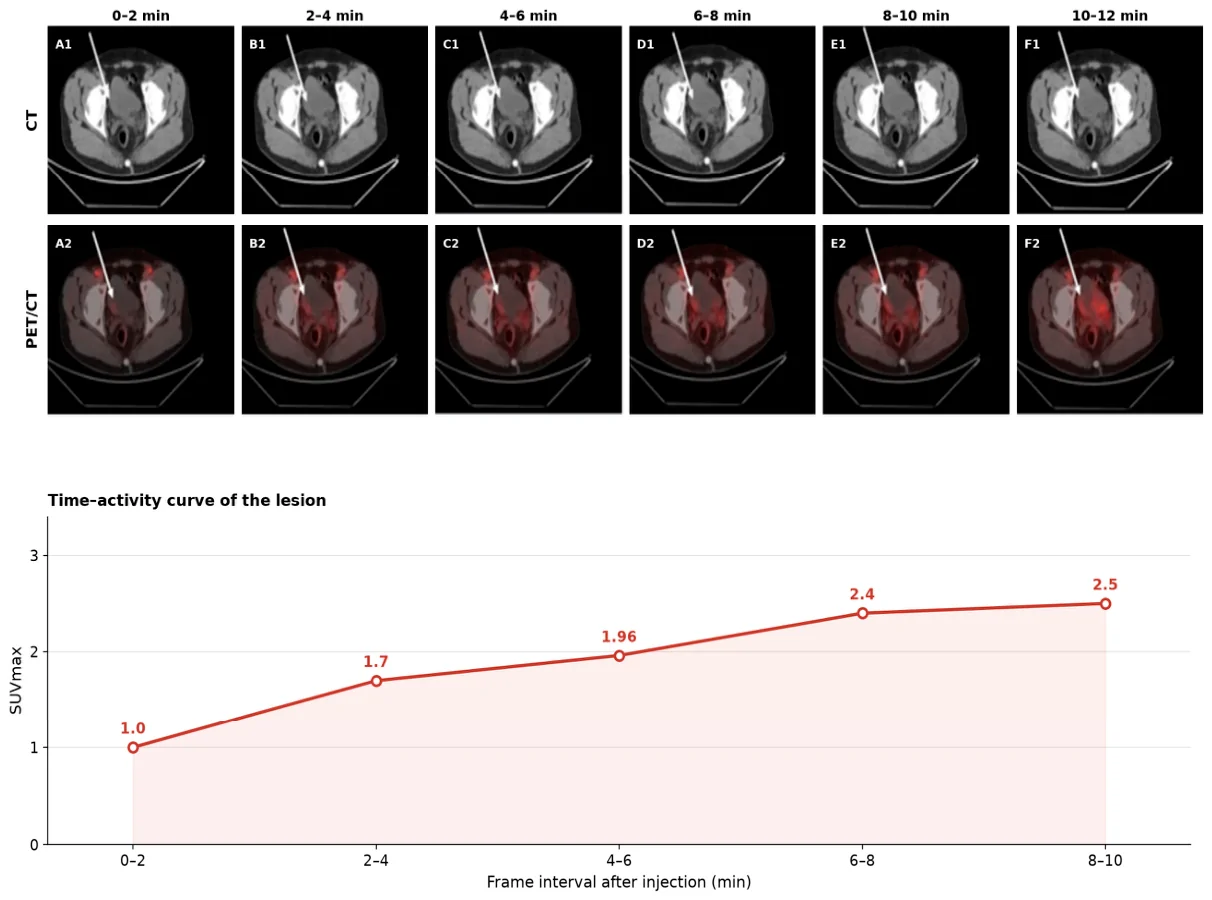
Representative Case—High-Grade Urothelial Carcinoma.

Representative cases illustrating the characteristic early dynamic FDG uptake patterns are presented in Figure 5 and Figure 6. Figure 5 demonstrates a low-grade non-invasive papillary urothelial carcinoma in a 66-year-old male patient, showing SUVmax values of 1.0, 1.6, and 2.8 at 0–2, 2–4, and 8–10 min, respectively, with a progressively increasing time-activity curve. Figure 6 shows a high-grade urothelial carcinoma in a 49-year-old male patient, with SUVmax values of 1.0, 1.7, and 2.5 at the corresponding time points, also exhibiting a progressive uptake pattern during the early dynamic phase. Both cases illustrate the early dynamic acquisition; their SUVmax values and time–activity curves were similar and are shown for illustration only.

Early dynamic phase non-contrast CT (top row, A1–F1) and fused PET/CT (bottom row, A2–F2) images of a 66-year-old male patient are presented. These images consist of six frames of two minutes each, covering a total duration of 12 min. The images were acquired in the transaxial plane. A tumor located on the right portion of the posterior wall of the urinary bladder, indicated by a white arrow, is observed. At 0–2 min (A2), the SUVmax of the tumor region was measured as 1.0; at 2–4 min (B2), the SUVmax was 1.6; and at 8–10 min (E2), the SUVmax was 2.8. The time–activity curve in the lower panel demonstrates that the tumor shows a progressively increasing FDG uptake pattern during the early dynamic phase. Histopathological examination after transurethral resection revealed a low-grade non-muscle-invasive bladder cancer in this area. The *x*-axis of the time–activity curve shows the frame interval (minutes after injection).

Early dynamic phase non-contrast CT (top row, A1–F1) and fused PET/CT (bottom row, A2–F2) images of a 49-year-old male patient are presented. These images consist of six frames, each lasting 2 min, covering a total duration of 12 min. The images were obtained in the transaxial plane. A tumor located in the posterior part of the right lateral bladder wall, indicated by a white arrow, is observed. At 0–2 min (A2), the SUVmax of the tumor region was measured as 1.0; at 2–4 min (B2), the SUVmax was 1.7; and at 8–10 min (E2), the SUVmax was 2.5. The time–activity curve shown in the lower panel demonstrates that the tumor exhibits a progressively increasing FDG uptake pattern during the early dynamic phase. Histopathological examination following transurethral resection revealed a high-grade urothelial carcinoma in this area. The *x*-axis of the time–activity curve shows the frame interval (minutes after injection).

## 4. Discussion

This prospective study investigated the diagnostic utility of multi-timepoint ^18^F-FDG PET/CT imaging for predicting tumor grade and pathological stage in patients with BC. Among 72 patients who underwent early dynamic (0–2, 2–4, 8–10 min), standard (60 min), and late (~120 min) imaging, the 0–2 min time point had the highest AUC for both high-grade tumors (AUC = 0.652) and muscle-invasive disease (AUC = 0.669) (Table 3, Figure 3). However, these values indicate only modest discrimination; the 95% CIs overlapped widely across time points, and that for muscle-invasive disease included 0.5 (0.489–0.849). High-grade tumors had significantly higher SUVmax than low-grade tumors at 0–2 min (median 1.50 vs. 1.00, *p* = 0.028) and at ~120 min (median 11.40 vs. 9.50, *p* = 0.045) (Table 2B, Figure 2D,F). The retention index did not discriminate between grades or stages (*p* = 0.484–0.540), suggesting that SUVmax at specific time points was more informative than the change over time in this cohort. SUVmax tended to be higher in T1 and T2 than in Ta tumors, but stage-wise differences were not statistically significant (Table 2A, Figure 2A–C). Together, these findings suggest an association between FDG uptake and tumor aggressiveness in BC, but they should be considered exploratory. The representative cases (Figure 5 and Figure 6) illustrate the acquisition protocol and do not by themselves demonstrate discrimination between low- and high-grade tumors.

Our findings align with and extend the existing body of evidence regarding FDG/PET imaging in BC, while providing preliminary insights into imaging timing. Sharma et al., in a prospective single-center study of 39 patients, reported that early dynamic SUVmax was significantly lower in non-invasive pTa tumors than in higher-stage tumors and tended to be higher in high-grade tumors (*p* = 0.144), which is broadly consistent with our findings [13]. The diagnostic performance of our early dynamic imaging protocol (sensitivity 53.7%, specificity 77.4% for high-grade prediction) is modest, and image-directed diagnostic pathways have so far relied on multiparametric MRI rather than FDG PET/CT [15]. A recent systematic review has reported variable sensitivity (23–89%) and specificity (81–100%) for ^18^F-FDG PET/CT in BC lymph node staging [16], highlighting the need for protocol optimization, which our multi-timepoint approach explores. Metabolic imaging appears advantageous over anatomical imaging particularly in lymph node assessment, where conventional CT relies solely on size criteria (>10 mm) and demonstrates poor sensitivity (26–46%) for detecting metastatic nodes [17,18]. Our study did not assess lymph node staging; it explored whether early dynamic metabolic parameters may help to characterize the primary tumor. The high specificity reported for nodal staging in previous studies suggests that ^18^F-FDG PET/CT is better suited to ruling in nodal disease when positive, though sensitivity limitations persist [19,20]. In our cohort, however, the PPV of the 0–2 min cut-off for muscle-invasive disease was only 35.0%. Importantly, recent prospective trials have demonstrated that ^18^F-FDG PET/CT changes treatment management in 10–31% of BC patients by detecting occult metastases missed by conventional imaging [21,22]. These findings collectively support metabolic imaging as a complement to anatomical staging, and our multi-timepoint protocol may offer a refinement in primary tumor characterization—a domain where previous studies primarily focused on nodal and distant metastases.

The present study addresses research gaps concerning the optimal timing of metabolic imaging and the usefulness of SUV parameters as predictors of tumor aggressiveness. Early dynamic SUVmax at 0–2 min showed a weak but significant correlation with tumor grade (ρ = 0.261, *p* = 0.027). For muscle-invasive disease, an SUVmax cut-off of 1.80 yielded a sensitivity of 58.3%, a specificity of 78.3%, and a negative predictive value of 90.4% (Table 3B). Because of the low prevalence of T2 disease (12/72) and the low sensitivity, this value does not allow muscle-invasive disease to be confidently excluded. The proposed cut-offs (SUVmax = 1.40 for high-grade and 1.80 for muscle-invasive disease at 0–2 min) were derived and evaluated in the same cohort and should be regarded as exploratory thresholds requiring external validation. Early dynamic SUVmax also correlated with tumor size (ρ = 0.424–0.440, *p* < 0.001), which may partly explain its associations with grade and stage and may reflect perfusion and blood-pool activity in the early phase rather than glucose metabolism alone. Because SUVmax in brief frames is sensitive to image noise, adjacent urinary activity, and partial-volume effects, early SUVmax should be regarded as a semi-quantitative index of early tracer delivery and uptake rather than a pure metabolic measure. These findings suggest that early dynamic imaging may capture biologically relevant tumor features, but this hypothesis needs confirmation in larger studies.

The novelty of this study lies in its multi-timepoint protocol, which evaluates ^18^F-FDG PET/CT at five time points within the same cohort, a dimension that has been little explored in the literature. While most prior studies focused on either standard (60 min) or delayed (120 min) imaging with diuretic protocols to overcome urinary interference [23,24,25], our results suggest that ultra-early dynamic imaging (0–2 min) may carry information about tumor grade and stage. This hypothesis-generating finding builds on the earlier dynamic imaging work of Sharma et al. [13] and warrants confirmation.

Our study has several limitations. The single-center design and the modest sample size (*n* = 72) limit generalizability; in particular, the SUVmax cut-offs were derived and evaluated in the same cohort and require validation in multicenter cohorts with diverse PET/CT scanners and reconstruction algorithms. Only 12 patients had T2 disease, and stage and grade were strongly related (all Ta tumors were low grade; all CIS and T2 tumors were high grade). Images were analyzed by a single reader, and interobserver agreement was not assessed. No formal a priori sample size calculation was performed, and the cut-offs were not subjected to internal validation (e.g., bootstrap resampling). Pathological staging relied on TURBT specimens, and repeat TURBT or radical cystectomy specimens were not systematically available to exclude understaging. In 6 of the 35 patients with multiple tumors, the PET/CT lesion could not be matched to the cystoscopic lesion and the most FDG-avid lesion was analyzed; this lesion may not be the one used for grading or staging, and a sensitivity analysis excluding these patients is reported in the Results. Early SUVmax measurements are affected by image noise in short frames, residual urinary activity, and partial-volume effects, and SUVmean, SUVpeak, and tumor-to-background ratios were not evaluated. Early SUVmax correlated with tumor size, and no multivariable adjustment was possible. Multiple time points and outcomes were tested without correction for multiplicity, and the lower confidence limit of the AUC for muscle-invasive disease was below 0.5. The adequacy of detrusor muscle sampling in TURBT specimens could not be verified systematically. Finally, the predominance of male patients (84.7%) mirrors epidemiological patterns but limits the assessment of sex-based differences.

## 5. Conclusions

This prospective study found that early dynamic ^18^F-FDG PET/CT imaging at 0–2 min had the highest, although modest, diagnostic performance among the evaluated time points for predicting tumor grade and muscle invasion in BC.

These preliminary findings are hypothesis-generating. The proposed SUVmax cut-offs are exploratory and require validation in larger multicenter studies before early dynamic imaging can be considered for clinical use in risk stratification.

## Figures and Tables

**Table 1:** Baseline Characteristics and Clinical Features of Study Population.

Characteristic	Overall (*n* = 72)	Ta (*n* = 26)	CIS (*n* = 11)	T1 (*n* = 23)	T2 (*n* = 12)	*p*-Value
Age (years), mean ± SD	64.8 ± 10.7	63.8 ± 9.7	63.6 ± 12.2	66.7 ± 10.2	64.0 ± 13.0	0.590 *
Gender, *n* (%)						0.956 †
Male	61 (84.7)	21 (80.8)	10 (90.9)	20 (87.0)	10 (83.3)	
Female	11 (15.3)	5 (19.2)	1 (9.1)	3 (13.0)	2 (16.7)	
Tumor size (cm), mean ± SD	2.84 ± 1.32	2.56 ± 1.16	2.92 ± 1.72	3.07 ± 1.34	2.92 ± 1.27	0.577 *
Tumor number, *n* (%)						0.734 †
Solitary	37 (51.4)	13 (50.0)	4 (36.4)	13 (56.5)	7 (58.3)	
Multiple	35 (48.6)	13 (50.0)	7 (63.6)	10 (43.5)	5 (41.7)	
Tumor grade, *n* (%)						<0.001 †
Low grade	31 (43.1)	26 (100.0)	0 (0.0)	5 (21.7)	0 (0.0)	
High grade	41 (56.9)	0 (0.0)	11 (100.0)	18 (78.3)	12 (100.0)	

* Kruskal–Wallis H test; † Chi-square test or Fisher’s exact test. Abbreviations: SD, standard deviation; Ta, non-invasive papillary carcinoma; CIS, carcinoma in situ; T1, tumor invades lamina propria; T2, tumor invades muscularis propria.

**Table 2:** ^18^F-FDG PET SUV Measurements by Pathological Stage and Tumor Grade.

**A. SUV Measurements by Pathological Stage**					
**SUV Parameter**	**Ta (*n* = 26)**	**CIS (*n* = 11)**	**T1 (*n* = 23)**	**T2 (*n* = 12)**	*p*-Value *
Early dynamic (0–2 min) SUVmax	1.00 (0.86–1.45)	1.00 (0.85–1.10)	1.40 (1.00–2.05)	1.80 (1.08–2.08)	0.069
Early dynamic (2–4 min) SUVmax	1.60 (1.20–2.22)	1.50 (1.20–1.95)	1.90 (1.29–2.80)	2.25 (1.45–2.65)	0.302
Early dynamic (8–10 min) SUVmax	3.49 (2.60–5.21)	2.70 (2.35–4.45)	3.90 (2.65–4.80)	3.85 (2.48–4.50)	0.697
Standard (60 min) SUVmax	6.38 (3.75–8.90)	5.80 (3.70–8.70)	7.70 (6.65–10.98)	7.58 (6.15–9.07)	0.138
Late imaging SUVmax	9.50 (4.79–12.47)	8.60 (5.30–13.50)	11.40 (9.70–17.59)	11.50 (10.22–13.20)	0.120
Retention index (%)	40.81 (25.48–68.07)	48.70 (39.85–70.40)	43.60 (22.10–63.04)	54.05 (37.50–79.88)	0.540
**B. SUV Measurements by Tumor Grade**					
**SUV Parameter**	**Low Grade (*n* = 31)**	**High Grade (*n* = 41)**	***p*-Value †**	**Effect Size ‡**	
Early dynamic (0–2 min) SUVmax	1.00 (0.83–1.27)	1.50 (1.00–2.00)	0.028 *	0.304	
Early dynamic (2–4 min) SUVmax	1.50 (1.15–1.95)	1.90 (1.36–2.70)	0.060	0.260	
Early dynamic (8–10 min) SUVmax	3.48 (2.50–5.31)	3.60 (2.50–4.70)	0.763	0.042	
Standard (60 min) SUVmax	6.70 (3.80–8.15)	7.90 (5.70–9.60)	0.096	0.231	
Late imaging SUVmax	9.50 (5.97–12.30)	11.40 (8.60–15.60)	0.045 *	0.279	
Retention index (%)	40.81 (25.95–67.61)	46.20 (32.40–77.50)	0.484	0.098	

(A): Values are median (interquartile range). * Kruskal–Wallis H test. (B): Values are median (interquartile range). † Mann–Whitney U test; ‡ Rank-biserial correlation coefficient. * *p* < 0.05 (shown in bold). Abbreviations: SUVmax, maximum standardized uptake value; IQR, interquartile range; min, minutes.

**Table 3:** Diagnostic Performance of SUV Parameters for Tumor Characterization.

**A. High-Grade Tumor Prediction**							
**SUV Parameter**	**AUC**	**95% CI**	**Cut-off**	**Sensitivity (%)**	**Specificity (%)**	**PPV (%)**	**NPV (%)**
Early dynamic (0–2 min)	0.652	0.526–0.778	1.40	53.7	77.4	75.9	55.8
Early dynamic (2–4 min)	0.630	0.502–0.758	2.10	46.3	77.4	73.1	52.2
Standard (60 min)	0.616	0.486–0.746	7.90	51.2	74.2	72.4	53.5
Late imaging (~120 min)	0.639	0.512–0.766	10.20	65.9	61.3	69.2	57.6
Retention index (%)	0.549	0.415–0.683	77.50	26.8	87.1	73.3	47.4
**B. Muscle-Invasive Disease (T2) Prediction**							
**SUV Parameter**	**AUC**	**95% CI**	**Cut-off**	**Sensitivity (%)**	**Specificity (%)**	**PPV (%)**	**NPV (%)**
Early dynamic (0–2 min)	0.669	0.489–0.849	1.80	58.3	78.3	35.0	90.4
Early dynamic (2–4 min)	0.630	0.448–0.812	2.20	58.3	70.0	28.0	89.4
Standard (60 min)	0.549	0.366–0.732	4.80	91.7	28.3	20.4	94.4
Late imaging (~120 min)	0.581	0.397–0.765	10.60	75.0	53.3	24.3	91.4
Retention index (%)	0.603	0.420–0.786	53.00	58.3	63.3	24.1	88.4

Abbreviations: AUC, area under the receiver operating characteristic curve; CI, confidence interval; PPV, positive predictive value; NPV, negative predictive value; SUVmax, maximum standardized uptake value; min, minutes. Cut-offs were determined by the Youden index and are exploratory. 95% CIs of the AUC were estimated with the DeLong method.

**Table 4:** Diagnostic Metrics of the 0–2 Minute SUVmax Cut-offs with 95% Confidence Intervals.

Outcome	Cut-Off	Sens., % (95% CI)	Spec., % (95% CI)	PPV, % (95% CI)	NPV, % (95% CI)	LR+ (95% CI)	LR− (95% CI)
High-grade tumor	1.40	53.7 (38.7–67.9)	77.4 (60.2–88.6)	75.9 (57.9–87.8)	55.8 (41.1–69.6)	2.38 (1.17–4.84)	0.60 (0.41–0.88)
Muscle-invasive (T2)	1.80	58.3 (32.0–80.7)	78.3 (66.4–86.9)	35.0 (18.1–56.7)	90.4 (79.4–95.8)	2.69 (1.37–5.31)	0.53 (0.27–1.05)

Abbreviations: CI, confidence interval; LR+, positive likelihood ratio; LR−, negative likelihood ratio; Sens., sensitivity; Spec., specificity; PPV, positive predictive value; NPV, negative predictive value. 95% CIs for proportions are Wilson score intervals and those for likelihood ratios were calculated with the log method; both were computed from the 2 × 2 tables corresponding to the values in Table 3 (high grade: TP 22, FN 19, TN 24, FP 7; T2: TP 7, FN 5, TN 47, FP 13). Cut-offs are exploratory.

## Data Availability

The datasets generated and analyzed during the current study are available from the corresponding author on reasonable request. Due to ethical restrictions and patient privacy considerations, the raw imaging data cannot be made publicly available but may be accessible for research purposes upon approval from the Mersin University Ethics Committee and with appropriate data sharing agreements in place.

## Abbreviations

The following abbreviations are used in this manuscript:

18F-FDG	Fluorine-18 fluorodeoxyglucose
AUC	Area under the curve
BC	Bladder cancer
BCG	Bacillus Calmette–Guérin
CI	Confidence interval
CIS	Carcinoma in situ
CT	Computed tomography
EAU	European Association of Urology
GLUT1	Glucose transporter 1
IQR	Interquartile range
ISUP	International Society of Urological Pathology
LN	Lymph node
MIBC	Muscle-invasive bladder cancer
MIP	Maximum Intensity Projection
MRI	Magnetic resonance imaging
NMIBC	Non-muscle-invasive bladder cancer
NPV	Negative predictive value
PET/CT	Positron emission tomography/computed tomography
PPV	Positive predictive value
ROC	Receiver operating characteristic
SD	Standard deviation
SUVmax	Maximum standardized uptake value
TNM	Tumor-node-metastasis
TURBT	Transurethral resection of bladder tumor
VOI	Volume of interest
WHO	World Health Organization

## References

[1] Omorphos N.P., Ghose A., Hayes J.D.B., Kandala A., Dasgupta P., Sharma A., Vasdev N. (2022). The increasing indications of FDG-PET/CT in the staging and management of Invasive Bladder Cancer. Urol. Oncol..

[2] Beyer T., Townsend D.W., Blodgett T.M. (2002). Dual-modality PET/CT tomography for clinical oncology. Q. J. Nucl. Med..

[3] Bacchiani M., Salamone V., Massaro E., Sandulli A., Mariottini R., Cadenar A., Di Maida F., Pradere B., Mertens L.S., Longoni M. (2023). Assessing the Performance of ^18^F-FDG PET/CT in Bladder Cancer: A Narrative Review of Current Evidence. Cancers.

[4] Macheda M.L., Rogers S., Best J.D. (2005). Molecular and cellular regulation of glucose transporter (GLUT) proteins in cancer. J. Cell. Physiol..

[5] Karantanis D., Allen-Auerbach M., Czernin J. (2012). Relationship among glycolytic phenotype, grade, and histological subtype in ovarian carcinoma. Clin. Nucl. Med..

[6] Roh J.L., Ryu C.H., Choi S.H., Kim J.S., Lee J.H., Cho K.J., Nam S.Y., Kim S.Y. (2007). Clinical utility of ^18^F-FDG PET for patients with salivary gland malignancies. J. Nucl. Med..

[7] Patronas N.J., Di Chiro G., Kufta C., Bairamian D., Kornblith P.L., Simon R., Larson S.M. (1985). Prediction of survival in glioma patients by means of positron emission tomography. J. Neurosurg..

[8] Swisher S.G., Erasmus J., Maish M., Correa A.M., Macapinlac H., Ajani J.A., Cox J.D., Komaki R.R., Hong D., Lee H.K. (2004). 2-Fluoro-2-deoxy-D-glucose positron emission tomography imaging is predictive of pathologic response and survival after preoperative chemoradiation in patients with esophageal carcinoma. Cancer.

[9] Ahuja V., Coleman R.E., Herndon J., Patz E.F. (1998). The prognostic significance of fluorodeoxyglucose positron emission tomography imaging for patients with nonsmall cell lung carcinoma. Cancer.

[10] Babjuk M., Burger M., Capoun O., Cohen D., Compérat E.M., Dominguez Escrig J.L., Gontero P., Liedberg F., Masson-Lecomte A., Mostafid A.H. (2022). European Association of Urology Guidelines on Non–muscle-invasive Bladder Cancer (Ta, T1, and Carcinoma in Situ). Eur. Urol..

[11] Witjes J.A., Bruins H.M., Cathomas R., Compérat E.M., Cowan N.C., Gakis G., Hernández V., Linares Espinós E., Lorch A., Neuzillet Y. (2021). European Association of Urology Guidelines on Muscle-invasive and Metastatic Bladder Cancer: Summary of the 2020 Guidelines. Eur. Urol..

[12] Anjos D.A., Etchebehere E.C., Ramos C.D., Santos A.O., Albertotti C., Camargo E.E. (2007). ^18^F-FDG PET/CT delayed images after diuretic for restaging invasive bladder cancer. J. Nucl. Med..

[13] Sharma A., Mete U.K., Sood A., Kakkar N., Gorla A.K., Mittal B.R. (2017). Utility of early dynamic and delayed post-diuretic ^18^F-FDG PET/CT SUVmax in predicting tumour grade and T-stage of urinary bladder carcinoma: Results from a prospective single centre study. Br. J. Radiol..

[14] Ceriani L., Suriano S., Ruberto T., Giovanella L. (2011). Could different hydration protocols affect the quality of ^18^F-FDG PET/CT images?. J. Nucl. Med. Technol..

[15] James N., Pirrie S., Liu W., Catto J., Jefferson K., Patel P., Hughes A., Pope A., Nanton V., Mintz H.P. (2024). Image directed redesign of bladder cancer treatment pathways: The BladderPath RCT. Health Technol. Assess..

[16] Aydh A., Abufaraj M., Mori K., Quhal F., Pradere B., Motlagh R.S., Mostafaei H., Karakiewicz P.I., Shariat S.F. (2020). Performance of fluoro-2-deoxy-D-glucose positron emission tomography-computed tomography imaging for lymph node staging in bladder and upper tract urothelial carcinoma: A systematic review. Arab J. Urol..

[17] Longoni M., Scilipoti P., Re C., Rosiello G., Nocera L., Pellegrino F., Basile G., de Angelis M., Quarta L., Burgio G. (2024). Use of 18F-fluoro-2-deoxy-d-glucose (^18^F-FDG) PET/CT for lymph node assessment before radical cystectomy in bladder cancer patients. BJU Int..

[18] Bertolaso P., Brouste V., Cazeau A.L., de Clermont-Gallerande H., Bladou F., Cabart M., Lefort F., Gross-Goupil M. (2022). Impact of 18 FDG- PET CT in the Management of Muscle Invasive Bladder Cancer. Clin. Genitourin. Cancer.

[19] Girard A., Rouanne M., Taconet S., Radulescu C., Neuzillet Y., Girma A., Beaufrere A., Lebret T., Le Stanc E., Grellier J.F. (2019). Integrated analysis of ^18^F-FDG PET/CT improves preoperative lymph node staging for patients with invasive bladder cancer. Eur. Radiol..

[20] Girard A., Dercle L., Vila-Reyes H., Schwartz L.H., Girma A., Bertaux M., Radulescu C., Lebret T., Delcroix O., Rouanne M. (2023). A machine-learning-based combination of criteria to detect bladder cancer lymph node metastasis on [18F]FDG PET/CT: A pathology-controlled study. Eur. Radiol..

[21] van Ginkel N., van Gennep E.J., Oosterbaan L., Greidanus J., Boellaard T.N., Wondergem M., Vis A.N., de Reijke T.M., van Rhijn B.W.G., Mertens L.S. (2023). Added Clinical Value of ^18^F-FDG-PET/CT to Stage Patients with High-Risk Non-Muscle Invasive Bladder Cancer Before Radical Cystectomy. Clin. Genitourin. Cancer.

[22] Kibel A.S., Dehdashti F., Katz M.D., Klim A.P., Grubb R.L., Humphrey P.A., Siegel C., Cao D., Gao F., Siegel B.A. (2009). Prospective study of [18F]fluorodeoxyglucose positron emission tomography/computed tomography for staging of muscle-invasive bladder carcinoma. J. Clin. Oncol..

[23] Harkirat S., Anand S., Jacob M. (2010). Forced diuresis and dual-phase ^18^F-fluorodeoxyglucose-PET/CT scan for restaging of urinary bladder cancers. Indian J. Radiol. Imaging.

[24] Yildirim-Poyraz N., Ozdemir E., Uzun B., Turkolmez S. (2013). Dual phase 18F-fluorodeoxyglucose positron emission tomography/computed tomography with forced diuresis in diagnostic imaging evaluation of bladder cancer. Rev. Esp. Med. Nucl. Imagen Mol..

[25] Li H., Wu H., Wang Q., Han Y., Wang Q. (2014). Value of dual-phase ^18^F-FDG PET/CT in preoperative staging of bladder cancer. Nan Fang Yi Ke Da Xue Xue Bao.

